# Climate change induced vulnerability and adaption for dengue incidence in Colombo and Kandy districts: the detailed investigation in Sri Lanka

**DOI:** 10.1186/s40249-020-00717-z

**Published:** 2020-07-23

**Authors:** Lahiru Udayanga, Nayana Gunathilaka, M. C. M. Iqbal, W. Abeyewickreme

**Affiliations:** 1 Department of Biosystems Engineering, Faculty of Agriculture & Plantation Management, Wayamba University of Sri Lanka, Makadura, Sri Lanka; 2 Department of Parasitology, Faculty of Medicine, University of Kelaniya, Ragama, Sri Lanka; 3 Plant and Environmental Sciences, National Institute of Fundamental Studies, Kandy, Sri Lanka; 4 Department of Parasitology, Faculty of Medicine, Sir John Kotelawala Defense University, Rathmalana, Sri Lanka

**Keywords:** Dengue, Climate change, Vulnerability, Sri Lanka

## Abstract

**Background:**

Assessing the vulnerability of an infectious disease such as dengue among endemic population is an important requirement to design proactive programmes in order to improve resilience capacity of vulnerable communities. The current study aimed to evaluate the climate change induced socio-economic vulnerability of local communities to dengue in Colombo and Kandy districts of Sri Lanka.

**Methods:**

A total of 42 variables (entomological, epidemiological, meteorological parameters, land-use practices and socio-demographic data) of all the 38 Medical Officer of Health (MOH) areas in the districts of Colombo and Kandy were considered as candidate variables for a composite index based vulnerability assessment. The Principal Component Analysis (PCA) was used in selecting and setting the weight for each indicator. Exposure, Sensitivity, Adaptive Capacity and Vulnerability of all MOH areas for dengue were calculated using the composite index approach recommended by the Intergovernmental Panel on Climate Change.

**Results:**

Out of 42 candidate variables, only 23 parameters (Exposure Index: six variables; Sensitivity Index: 11 variables; Adaptive Capacity Index: six variables) were selected as indicators to assess climate change vulnerability to dengue. Colombo Municipal Council (CMC) MOH area denoted the highest values for exposure (0.89: exceptionally high exposure), sensitivity (0.86: exceptionally high sensitivity) in Colombo, while Kandy Municipal Council (KMC) area reported the highest exposure (0.79: high exposure) and sensitivity (0.77: high sensitivity) in Kandy. Piliyandala MOH area denoted the highest level of adaptive capacity (0.66) in Colombo followed by Menikhinna (0.68) in Kandy. The highest vulnerability (0.45: moderate vulnerability) to dengue was indicated from CMC and the lowest indicated from Galaha MOH (0.15; very low vulnerability) in Kandy. Interestingly the KMC MOH area had a notable vulnerability of 0.41 (moderate vulnerability), which was the highest within Kandy.

**Conclusions:**

In general, vulnerability for dengue was relatively higher within the MOH areas of Colombo, than in Kandy, suggesting a higher degree of potential susceptibility to dengue within and among local communities of Colombo. Vector Controlling Entities are recommended to consider the spatial variations in vulnerability of local communities to dengue for decision making, especially in allocation of limited financial, human and mechanical resources for dengue epidemic management.

## Background

Dengue has become a challenge for both health and economic sectors in the world with an estimated infection rate of 50–100 million infections per year [1]. Many parts of the world, including tropical, sub-tropical countries and even in temperate countries (such as Europe and North America), have been recognized to be at a risk for dengue, especially with global warming, unplanned urbanization, co-circulation of different dengue virus serotypes (DEN 1, DEN 2, DEN 3 and DEN4), international trade and transportation [2–7]. Therefore, urban and suburban environments in many tropical and sub-tropical regions of the world remain under a high risk of severe dengue outbreaks [8–10]. The first dengue incidence in Sri Lanka was reported in 1965, while the worst dengue epidemic has caused a total of 186 101 dengue cases with more than 350 deaths in 2017. Meanwhile, approximately 105 049 suspected dengue cases were reported in 2019 as the second worst epidemic [11].

A variety of factors such as characteristics of the susceptible populations, vector ecology, mosquito density, local environmental conditions (meteorological parameters, land use, vegetation and elevation) and circulating serotype(s) of the virus influence the incidence of dengue epidemics [9, 10]. Recent changes in climatic conditions and development of insecticide resistance pose a greater threat from vector borne diseases [12–14]. Changes in climate could result in direct impacts on the growth and development of mosquito vectors that transmit dengue, resulting in an elevated risk of dengue upon vulnerable communities.

Often, climate acts as a major barrier in restricting the geographic distribution of vector borne diseases, through influencing the survival of mosquito vectors [15, 16]. On the other hand, numerous models have predicted that climate changes would increase the geographic distribution and potential risk of dengue incidence [17]. Such alarmingly severe dengue epidemics impose a serious challenge to the Vector Controlling Entities (VCE), which attempt to manage dengue epidemics. Similar to many developing countries, Sri Lanka also focuses mainly on vector control and management in dengue control. However, numerous limitations in human, mechanical and financial resources influence negatively on the success of dengue epidemic management [18]. Therefore, recognition of the potential risk factors that govern the incidence and severity of dengue epidemics, forecasting dengue outbreaks, assessing vulnerability, implementing proactive programmes to reduce existing vulnerabilities and improving resilience capacity of the vulnerable communities are some of the key strategies to ensure the success of dengue epidemic management [17–20].

Regardless of temporal and spatial variations in nature, relationship among meteorological parameters with dengue epidemics has been well evidenced. In general, temperature has denoted a direct influence on reproduction, biting behavior, distribution patterns, survival rate and extrinsic incubation period (EIP) of the *Aedes* mosquitoes, thereby influencing the incidence and spread of dengue epidemics [21–23]. On the other hand, rainfall also has a positive impact on abundance of *Aedes* vectors via increasing the abundance of potential vector breeding sites [24–26]. Relative humidity is another important meteorological factor that directly influence the mating patterns, egg laying, feeding patterns (duration and frequency) and longevity of adult mosquitoes [27–29]. Any change in the average weather patterns, which may be recognized as a climate change, could result in significant influences on the incidence, spread and severity of dengue epidemics [17, 20, 30].

The degree to which a system or a population remains prone to or incapable of dealing adverse impacts resulting from climate change is understood as vulnerability [31]. According to Smit and Wandel, vulnerability is expressed as a function of three sub-indices namely exposure, sensitivity and adaptive capacity [32]. The degree, duration or frequency of considering a stress factor imposed on a system is understood as exposure, while the extent to which the considering system is influenced by the stress factor is defined as sensitivity. On the other hand, adaptive capacity is defined as the ability of a system to withstand the stress in response to actual or expected climatic stimuli or their effects, moderating the harm or exploiting beneficial opportunities [32–35]. Both exposure and sensitivity shares a positive association with the vulnerability, accounting for the potential impact. Meanwhile, adaptive capacity is the ability of the system to cope with the potential impacts, indicating a negative relationship with the vulnerability [33–35]. The concept of vulnerability is a widely accepted concept that is heavily used in disaster management aspects and in climate change related disciplines. Often, climate change vulnerability is assessed to understand the potential risk imposed by the climate and other attributes on the considering system and to identify the key areas to be focused to enhance resilience of the system against changes in the climate, especially in the case of public health [17, 36–39].

Despite the variations in methodologies used, such as statistical and Geographic Information Systems (GIS) based mapping, majority of these studies have not been conducted based on a clear conceptual vulnerability framework, restricting the overall applicability of the methodology and comparability of results [17, 37, 38]. Almost all these studies have limited their focus up to recognition of risk factors, risk mapping, risk prediction or modelling and development of dengue surveillance systems [17, 34, 39–41], while vulnerability of dengue has been limitedly studied. In the context of developing countries, evaluation of vulnerability would be immensely valuable for the government entities to assess the health burden of dengue and to plan long-term strategies to improve the resilience of local communities to dengue in the face of climate change [17, 38].

The current study intends to address this knowledge gap by evaluating the spatial and socioeconomic vulnerability of the populations residing in Colombo and Kandy districts of Sri Lanka to dengue, through a composite index approach recommended by the Intergovernmental Panel on Climate Change (IPCC), thereby allowing the VCE to estimate the burden of dengue, key areas to be monitored for susceptibility and to identify intervention options for reducing susceptibilities and strengthening resilience to dengue in Sri Lanka.

## Methods

### Study area

**Fig. 1:**
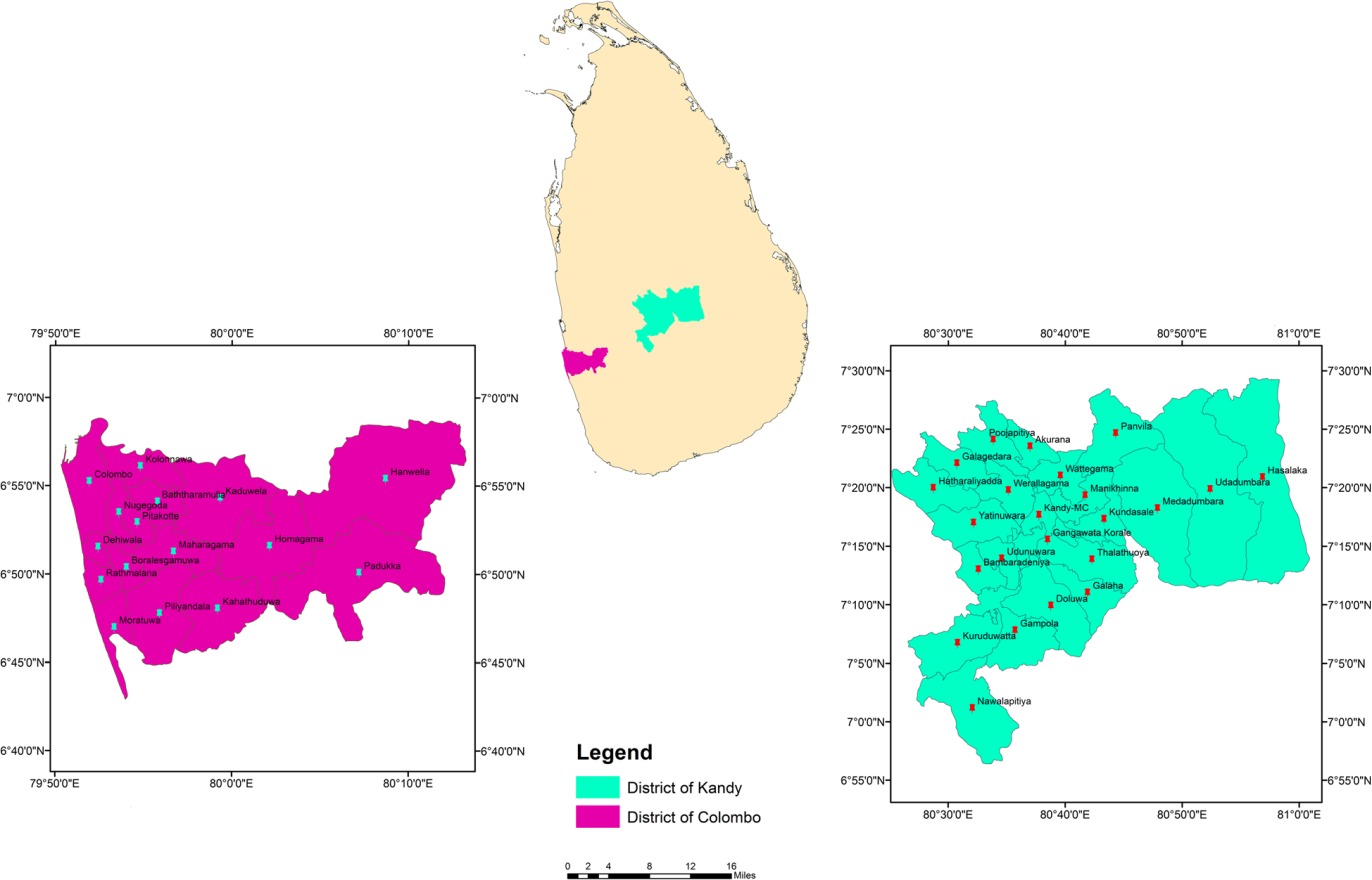
Location of the studied MOH areas within Sri Lanka

Districts of Colombo (6.70° to 6.98°N and 79.83° to 80.22°E) and Kandy (6.93° to 7.50°N and 80.43° to 81.04°E) in Sri Lanka, were selected as the study areas (Fig. 1). Of 105 049 suspected dengue cases reported in 2019, Colombo and Kandy districts have accounted for 19.7% (*n* = 20 718) and 8.5% (*n* = 8940), becoming the first and fourth high risk areas for dengue in Sri Lanka [11]. Colombo district is subdivided into 16 Medical Officers of Health (MOH) areas to facilitate the monitoring and management of health-related issues. A total population of 2 309 809 resides within Colombo, resulting in a population density of 3305 people per km^2^ [42]. The climate in Colombo is typically tropical with an average temperature of 28 °C to 32 °C. Heavy rains occur during the monsoon seasons from South-West monsoon (May to August) and North-East monsoon (October to January), providing a total rainfall that exceeds 2500 mm per year. Relative Humidity (RH) varies from 70% during the day to 90% at night [42]. In the case of Kandy, the total land extent of 1940 km^2^ hosts a population of 1 369 899 people, is subdivided into 23 MOH areas [43]. The average cumulative rainfall received by Kandy is approximately 2500 mm per year, with an average temperature of 20–22 °C throughout the year.

### Data collection

Entomological findings (Premises Index [PI], Breteau Index [BI] and Container Index [CI]) for the period of January, 2012 to December, 2019, were collected from the relevant MOH offices along with the number of reported dengue cases. As meteorological parameters, monthly total rainfall, minimum and maximum temperature and mean relative humidity of the study areas relevant to the above period of study, were obtained from the Department of Meteorology, Colombo, Sri Lanka. In addition, digital topographical information (land use, transport, hydro, building, terrain and administration) of the study areas were collected from the Department of Survey, Colombo, Sri Lanka at 1:50 000 scale.

The following socio-economic parameters; total population, percentage of males and females, percentage of population belonging to different age groups (below 20 years, 21–40 years, 41–60 years, 60–80 years and above 80 years), percentage population breakdown based on educational levels (illiterate, primary education completed, secondary education completed, General Certificate of Education Advanced Level [GCE A/L, *a local examination prior university entrance*] completed and above), percentage of population indicating different waste disposal practices (collected by Municipal Councils or Pradeshiya Sabha [a regional administrative authority], open dumping, burying, burning, improper disposal and composting) and percentage of population with access to different communication facilities (television, radio, mobile phones, fax and computers etc.) were acquired from the Department of Census and Statistics, Colombo, Sri Lanka at the Grama Niladhari Division (GND) level corresponding to the above study period.

### Data processing

All the collected socio-economic parameters were rearranged at the MOH level by combining the GND level data appropriately. In case of topographical information, land use maps were developed by using ArcGIS (Esri, United States, version 10.2) software package and the extent of different land use types (built environment, home gardens, tea, paddy, coconut, rubber, waterbodies, forests, scrublands, marshes and swamps, grasslands, quarries and barren lands etc.) were calculated with the geo-calculator tool. The meteorological stations were created as a shape file and continuous raster files depicting the spatial variation of different meteorological parameters (rainfall, temperature and relative humidity) were developed with a spatial resolution of 500 m using spatial interpolation tools in ArcMap. Subsequently, centroids of the MOH areas were developed and the values of the relevant meteorological parameters at each centroid were extracted from above developed raster layers by using the “Extract by Point” tool.

### Vulnerability assessment

All collected variables were considered as potential indicators for the vulnerability assessment as highlighted by the Gesellschaft für Internationale Zusammenarbeit (GIZ), referred to as indicator approach [44]. Potential indicators that represents the three domains (exposure, sensitivity and adaptive capacity) of climate change vulnerability of dengue incidence were recognized based on literature and expertise knowledge, separately. All the potential variables of each domain were standardized (followed by square root transformation, where necessary) and Principal Components Analysis (PCA) was used for the identification of the most reflective and non-correlated indicators of each domain [45].

The Kaisere Mayere Olkin (KMO) sampling adequacy and Bartlett’s sphericity tests were used to ensure the suitability of the variables for PCA analysis. Kaiser’s rule of thumb, which retains the Principle Components (PC) with eigenvalues > 1.0 was followed in retaining the most-significant PCs for further analysis, while considering the proportion of the total variation accounted by the PCs [44]. Variation max standardizing method with Kaiser normalization was used for the construction of the rotated component matrix, while suppressing candidate indicators with coefficients < 0.70, to retain the most significant, representative and non-correlated variables. The indicators retained in the rotated matrix were selected as the candidate variables in each domain. For such indicators, the eigenvalues of PC (*E*) and the loading coefficients (*β*) were recorded. The Principal Component Analysis (PCA) combined with a factor analysis was used to draw out the representative indicators for each domain and to calculate the reflective weights for each indicator in each domain. SPSS (IBM, United States, version 23) was used for all the statistical treatments.

Since, different indicators that have been selected as candidates exist in different units and scales, a standard normalization procedure was followed to transform the indicator values of the MOH areas into unitless uniform scales. Equation 1 was used for the indicators that indicated a positive relationship with the domain, while Eq. 2 was used for the rest of the candidate indicators [44].

1 $$ {x}_{ij}=\frac{X_{ij}-\mathit{\operatorname{Min}}\left\{{X}_{ij}\right\}}{\mathit{\operatorname{Max}}\left\{{X}_{ij}\right\}-\mathit{\operatorname{Min}}\left\{{X}_{ij}\right\}} $$

2 $$ {x}_{ij}=\frac{\mathit{\operatorname{Max}}\left\{{X}_{ij}\right\}-{X}_{ij}}{\mathit{\operatorname{Max}}\left\{{X}_{ij}\right\}-\mathit{\operatorname{Min}}\left\{{X}_{ij}\right\}} $$

Where, *x*_*ij*_ is the normalized value of indicator (*j*) with respect to MOH (*i*). *X*_*i*_ is the actual value of the indicator with respect to MOH (*i*). *Min*{*X*_*j*_} and *Max*{*X*_*j*_} are the minimum and maximum values with respect to indicator (*j*) among all considered DSDs. After normalization the sub-indices (*I*_*i*_) relevant for the three domains were calculated for each MOH based on the normalized values of the relevant indicators by using the Eq. 3 [44].

3 $$ {I}_i=\frac{\sum \left[{\beta}_jX\ {E}_jX\ {x}_{ij}\right]\ \left(j=1,2,3\dots .n\right)}{\sum \left[{\beta}_jX\ {E}_j\right]} $$

Where *I*_*i*_ is the sub-index (Exposure, Sensitivity or Adaptive Capacity); *i* is the MOH area under consideration; *E*_*j*_ is the eigenvalues of PC, which has the highest loading coefficient of the considering indicator (*j*); *β*_*j*_ is the highest loading coefficient of indicator *j* obtained from the rotated PC matrix, and *x*_*ij*_ is the normalized value of value of indicator (*j*).

After calculation of the three sub-indices as Exposure Index (EI), Sensitivity Index (SI) and Adaptive Capacity Index (AI), the vulnerability of dengue incidence to climate change was calculated for all the MOH areas as indicated in the Eq. 4 [43].

4 $$ Vulnurability\ Index\ (VI)=\frac{\left[ EI+ SI- AC\right]}{3} $$

Five vulnerability categories were defined for all the sub-indices based on the index score as, “Very Low” (0–0.20), “Low” (0.21–0.40), “Moderate” (0.41–0.60), “High” (0.61–0.80) and “Exceptionally High” (0.81–1.00) [43]. The sub index values and VI scores of the MOH areas were mapped by using ArcMap, to represent the spatial variations of climate change vulnerability of dengue in the districts of Colombo and Kandy.

### Ethical aspects

Ethical approval was obtained from the Ethics Review Committee of the Faculty of Medicine, University of Kelaniya (P/155/10/2015). The confidentiality of the acquired data was maintained throughout the study.

## Results

### Exposure index

Only two PCs that had eigenvalues > 1 survived the extraction and rotation steps in the PCA. In total, the retained PCs accounted for 83.0% of the total variation (Table 1). Among the eight candidate variables that were considered, only six variables, namely, monthly cumulative rainfall, average temperature, average relative humidity, number of reported dengue cases, average BI and PI, were retained in the two PCs with loading coefficients > 0.70 (Table 1). Meteorological parameters were loaded on to the first PC that accounted for 68.9% of total variation, while reported dengue cases, BI and PI were loaded on to the other.

**Table 1:** Loadings of the factors considered for exposure after rotation of the component matrix

Factors	Principal components	
	1	2
Rainfall	0.967	
Average Temperature	0.852	
Relative Humidity	0.886	
Reported Dengue Cases		0.857
Breteau Index (BI)		0.956
Premise Index (PI)		0.927
Variation explained by each PC after rotation	68.9	18.1

Extraction Method: Principal Component Analysis; Rotation Method: Varimax with Kaiser Normalization; Factors with loading coefficients < 0.70 have been suppressed

**Fig. 2:**
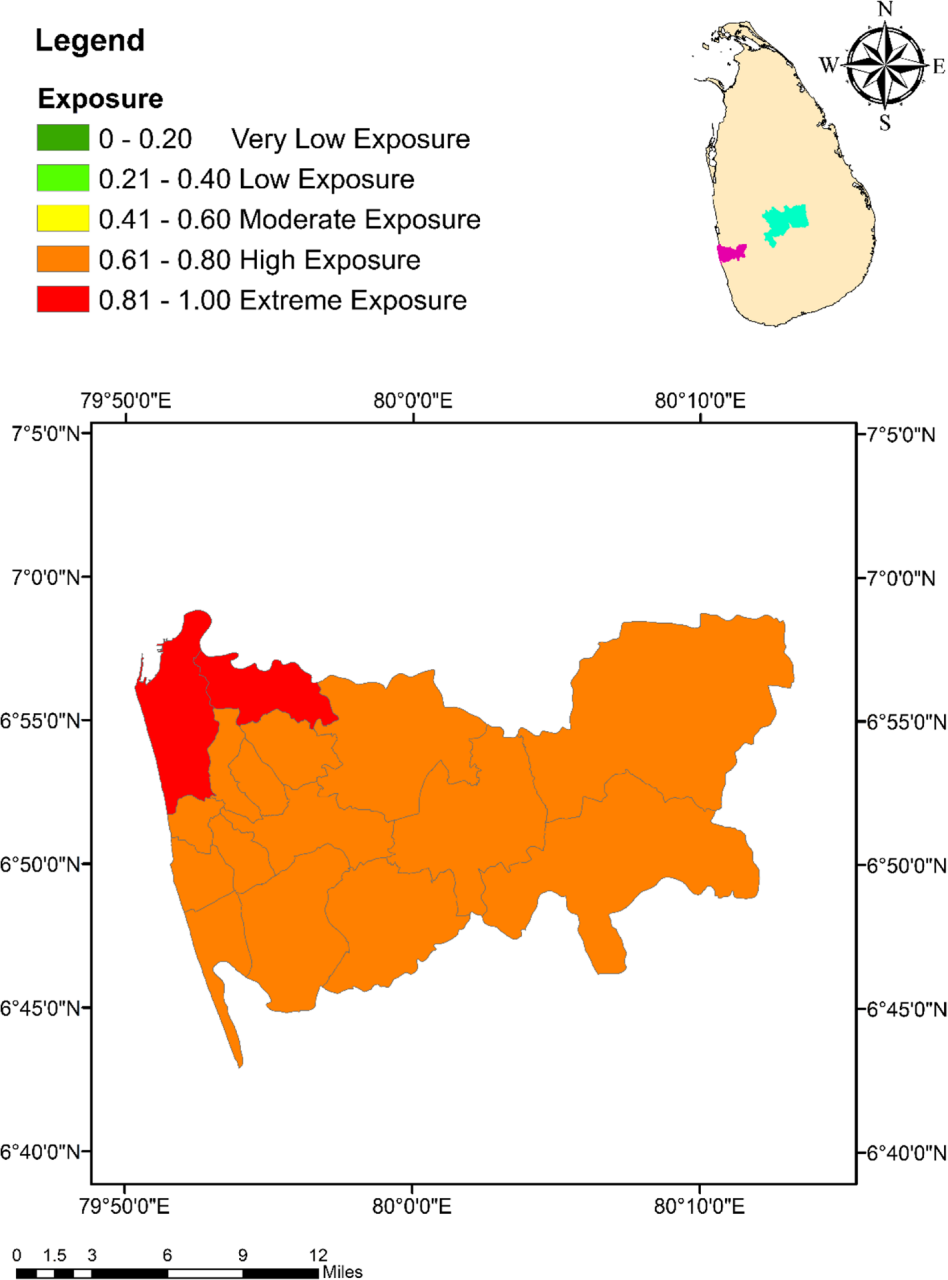
Spatial variation of the Exposure Index values among the MOH areas within the district of Colombo

**Fig. 3:**
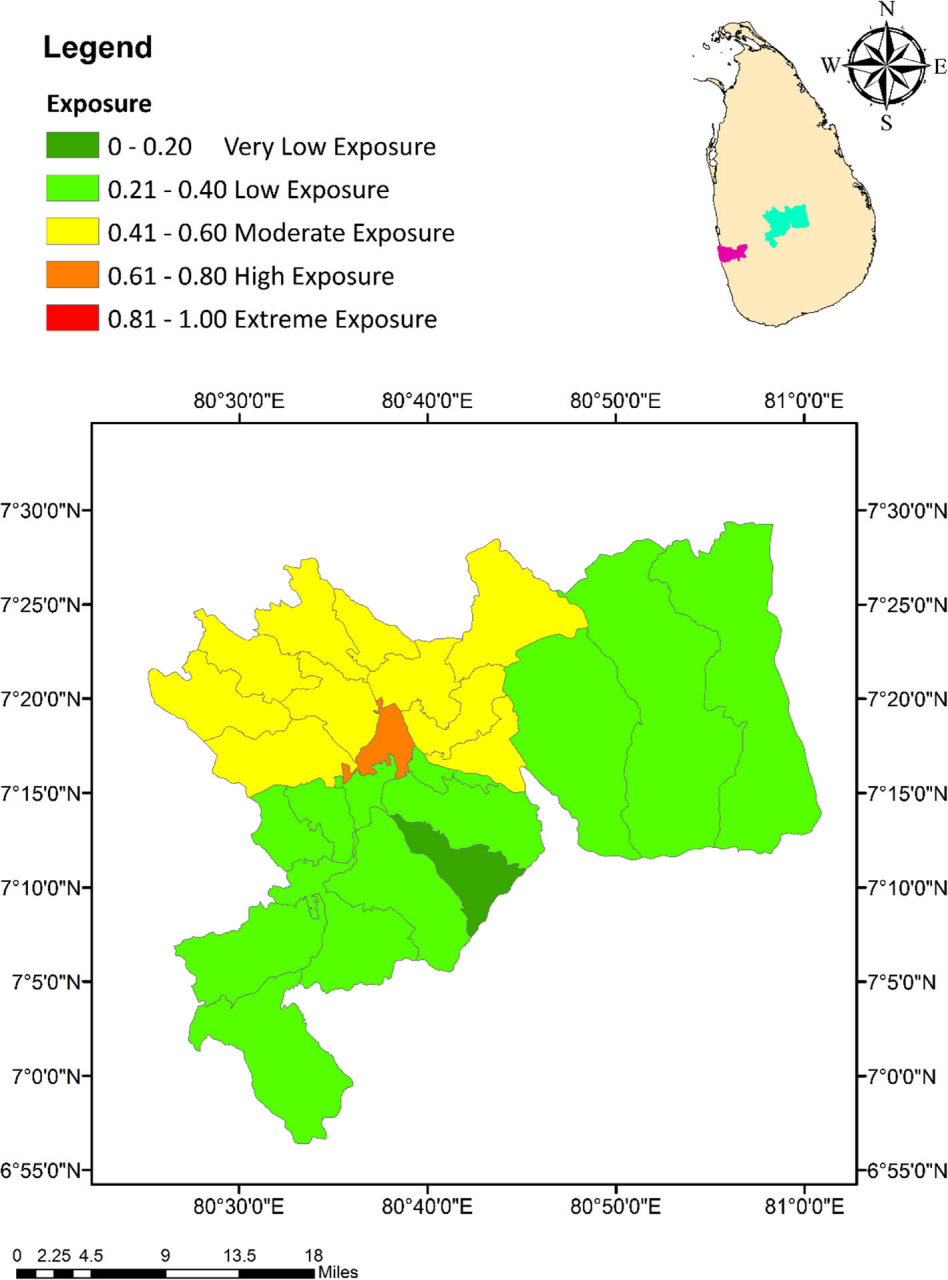
Spatial variation of the Exposure Index values among the MOH areas within the district of Kandy

Based on the composite index approach, the MOH areas in Colombo had a relatively higher level of exposure for climate change than the MOH areas in Kandy. The highest exposure level of 0.89 (exceptionally high) in Colombo was expressed by the Colombo Municipal Council MOH area, while the lowest (0.71) was observed from Hanwella/Avissawella (Fig. 2). In the case of Kandy, Kandy Municipal Council areas had the highest exposure of 0.79. With an exposure value of 0.19, Galaha MOH area indicated the lowest degree of exposure of dengue to climate change (Fig. 3).

### Sensitivity index

Among 25 variables, only 11 variables loaded on to five PCs were retained after the rotation of PCs, and the rotation of the component matrix, without being suppressed (Table 2). The PC1, included the total population, percentage area covered by built environment and the forests accounting for 32.6% of total variation. Meanwhile, percentage of males and females, percentage of population belonging to the age group of 21–40 years and above 60 years constituted the PC2. Total households in the MOH areas formed the PC3, while percentage of households practicing composting constructed the PC4. Finally, waste collection by the Municipal Council or Pradeshiya Sabha and percentage of houses that burn waste were included in PC5 (Table 2). In total, all the five PCs accounted for 85.3% of the total variation.

**Table 2:** Loadings of the factors considered for sensitivity after rotation of the component matrix

Factors	Principal components (PC)				
	1	2	3	4	5
Total Population	0.947				
Percentage of Males		0.794			
Percentage of Females		-0.709			
Percentage of population belonging to the age group of 21–40 years		0.708			
Percentage of population above 60 years		0.837			
Total Households			0.886		
Percentage of households disposing waste via Municipal Council					-0.844
Percentage of households disposing that burn waste					-0.781
Percentage area covered by Built Environment	0.763				
Percentage of households practicing Composting				-0.702	
Percentage area covered by Forests	0.809				
Variation explained by each PC after rotation	32.6	23.4	10.9	9.6	8.9

Extraction Method: Principal Component Analysis; Rotation Method: Varimax with Kaiser Normalization; Factors with loading coefficients < 0.70 have been suppressed

**Fig. 4:**
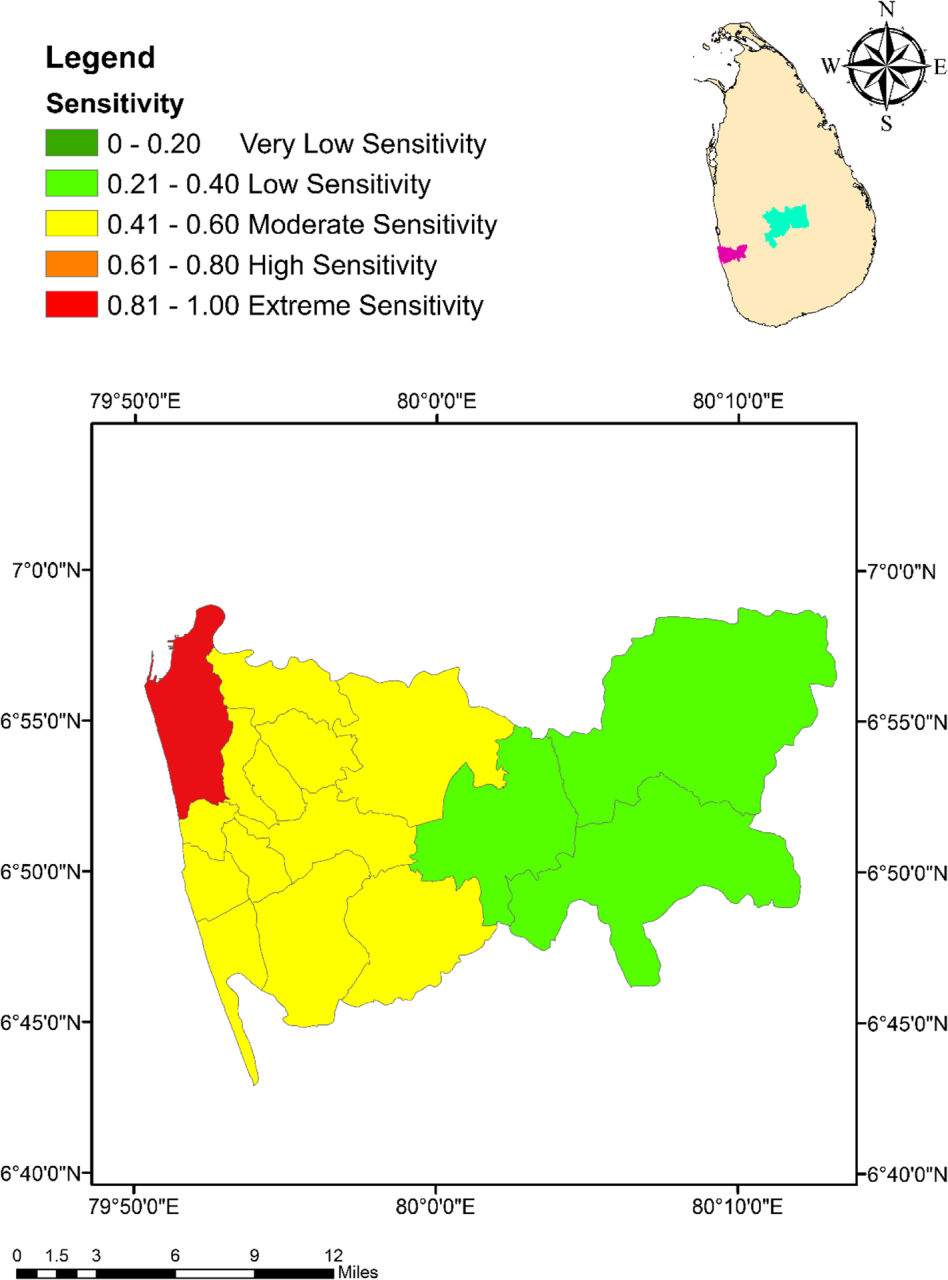
Spatial variation of the Sensitivity Index values among the MOH areas within the district of Colombo

**Fig. 5:**
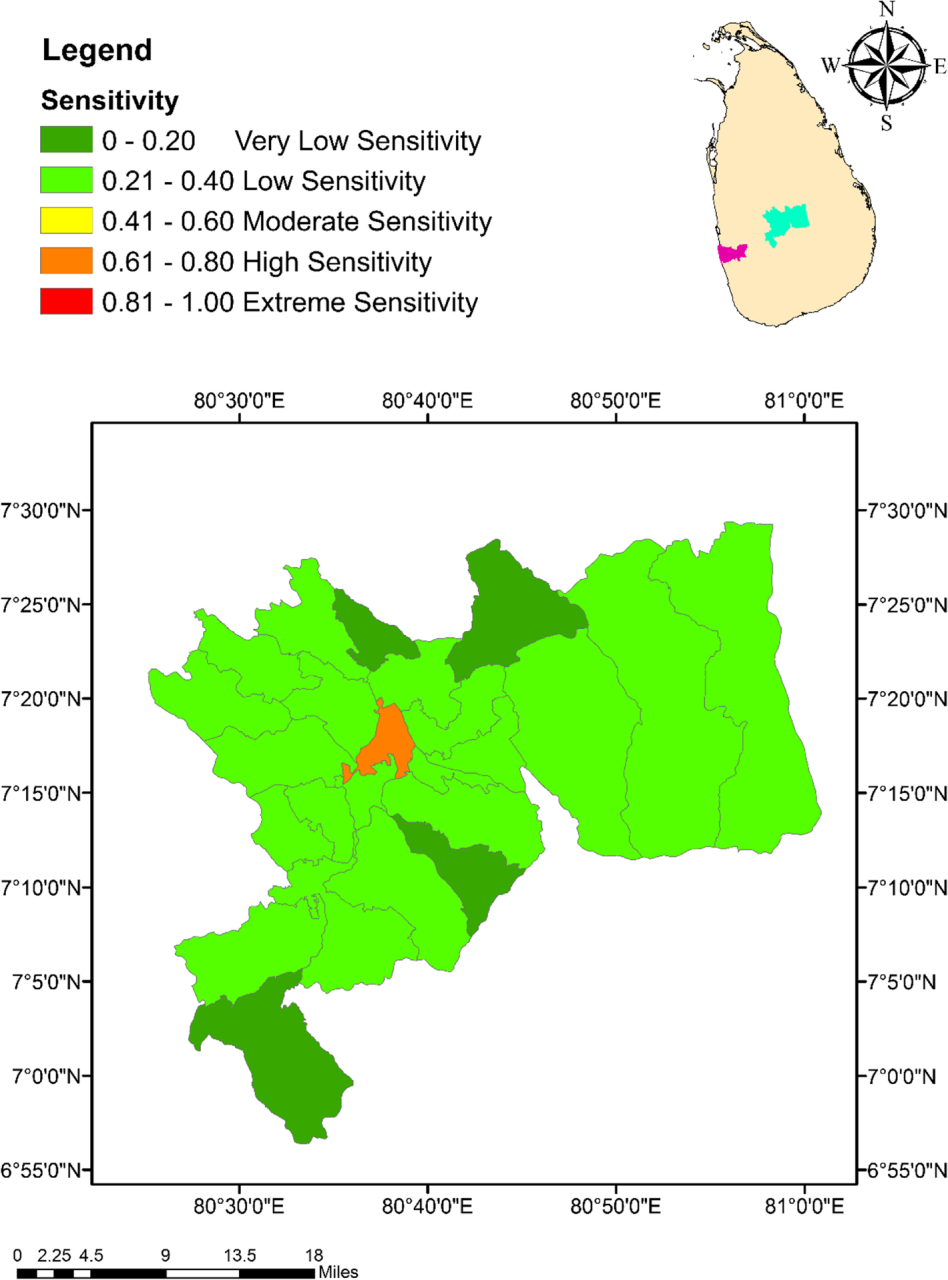
Spatial variation of the Sensitivity Index values among the MOH areas within the district of Kandy

Similar to the Exposure Index (EI), the MOH areas in Colombo had a relatively higher level of sensitivity for climate change than the MOH areas in Kandy. With a sensitivity of 0.86 (exceptionally high sensitivity), Colombo Municipal Council MOH area denoted the highest degree of sensitivity, while Homagama had the lowest numerical value for sensitivity (0.38; low sensitivity) as indicated in Fig. 4. The highest sensitivity level of 0.77 (high sensitivity) in Kandy was expressed by Kandy Municipal Council area, while Galaha MOH area indicated the lowest sensitivity value of 0.15 (very low sensitivity) as indicated in Fig. 5.

### Adaptive capacity index

**Fig. 6:**
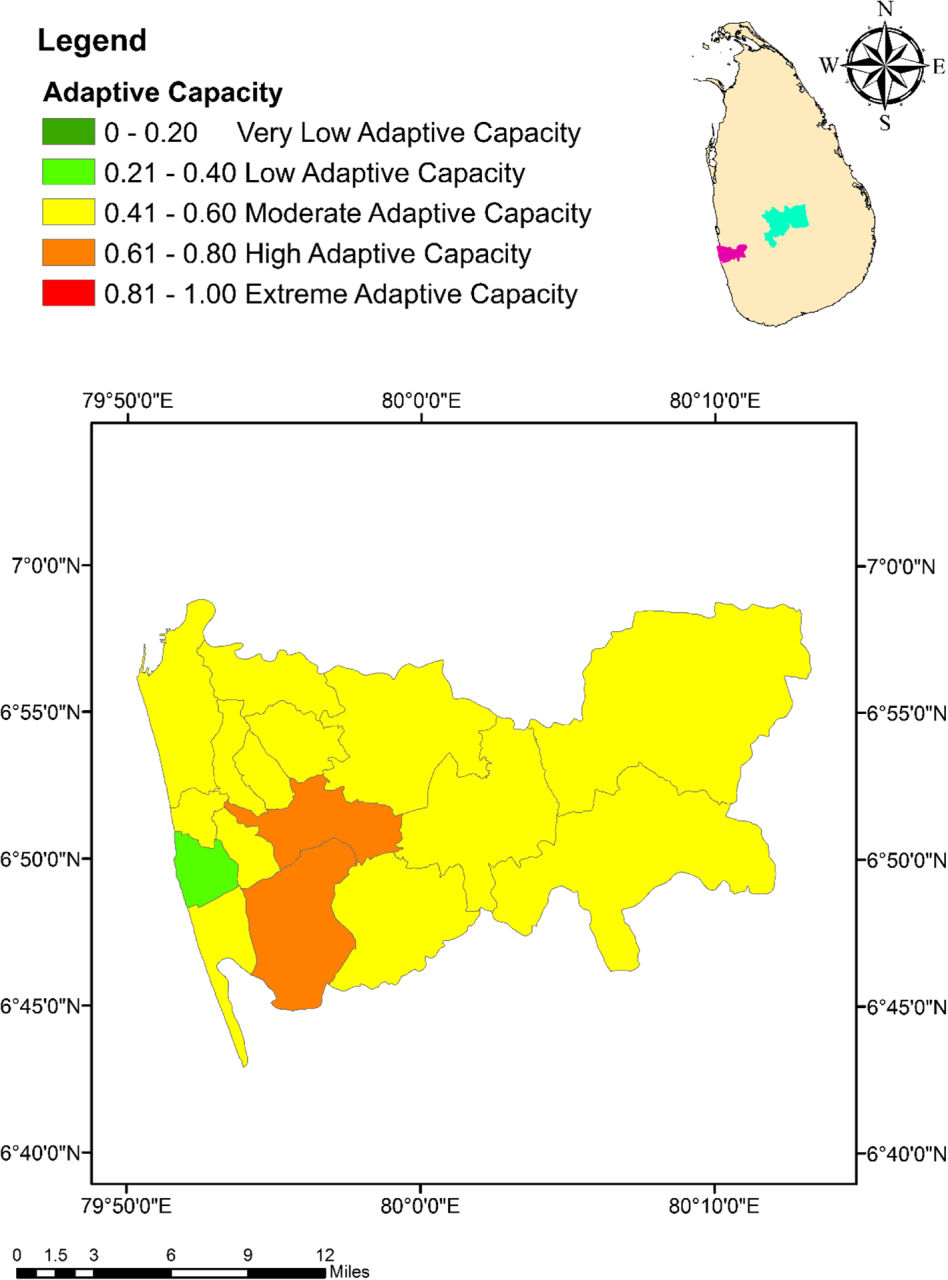
Spatial variation of the Adaptive Capacity Index values among the MOH areas within the district of Colombo

**Fig. 7:**
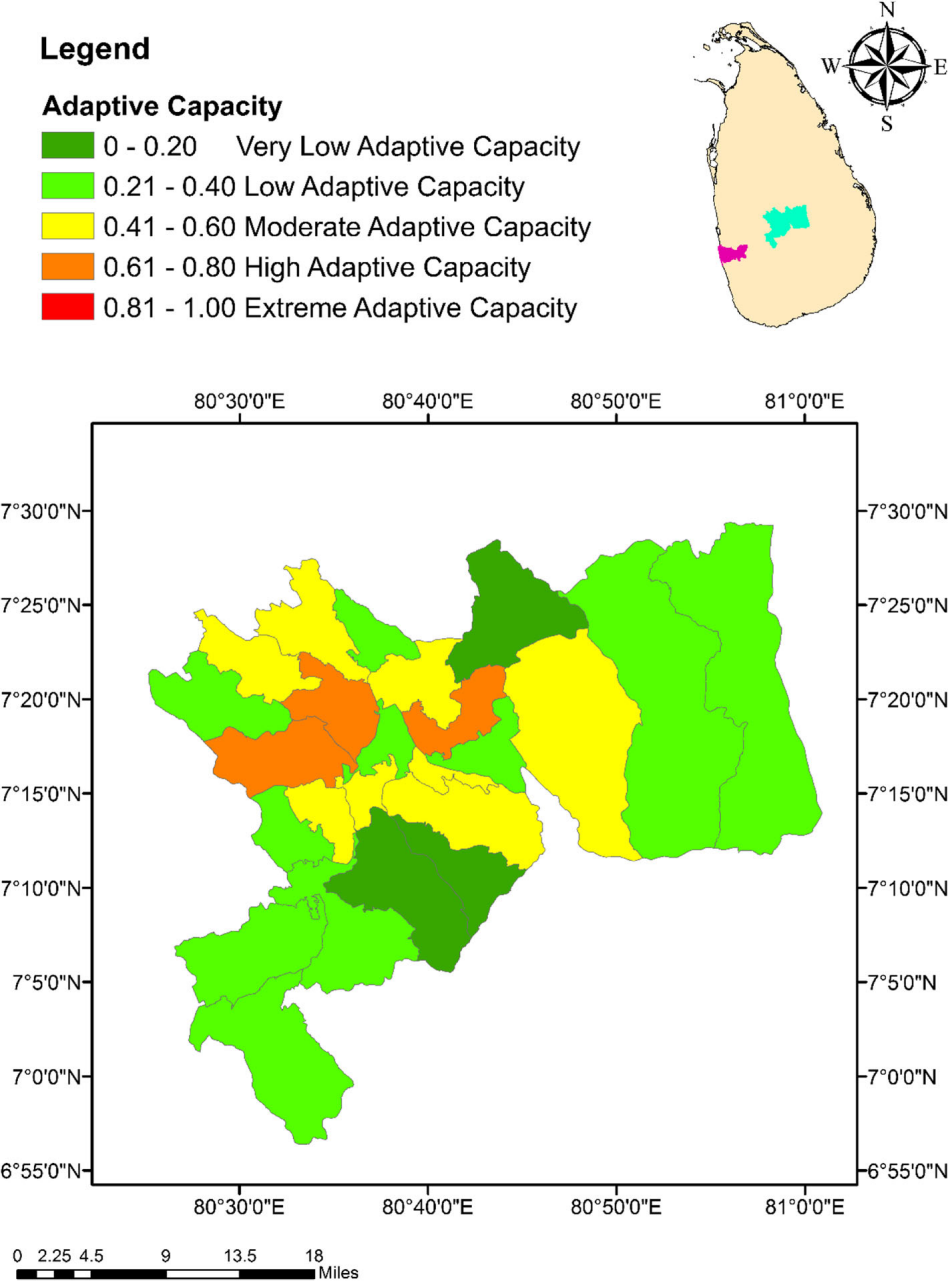
Spatial variation of the Adaptive Capacity values among the MOH areas within the district of Kandy

As indicated in Table 3, only six variables survived the PCA analysis, out of the nine candidate variables that were considered for adaptation capacity of dengue against climate change. Percentage of households with access to television and radios were loaded onto the PC1 accounting for 35.0% of the total variation. Percentage of population with no formal education and with education above GCE Ordinary Level (O/L) constituted the PC2, while the number of medical officers and Public Health Inspectors (PHI) for 1000 residents in a MOH area formed the PC3. In general, a total of 84.0% of the total variation was accounted by the retaining three PCs. Piliyandala MOH area was characterized with the highest level of adaptive capacity (0.66) in the district of Colombo, while Menikhinna had the highest value (0.68) in Kandy. In contrast, Rathmalana (0.28) and Panwila (0.16) showed the lowest degree of adaptive capacity in the districts of Colombo and Kandy, respectively (Figs. 6 and 7).

**Table 3:** Loadings of the factors considered for adaptive capacity after rotation of the component matrix

Factors	Component		
	1	2	3
Percentage of population without any education		-0.933	
Percentage of population with education above O/L		0.930	
Percentage of houses with radios	0.705		
Percentage of houses with television	0.728		
Number of doctors in the MOH area			0.902
Number of PHI officers in the MOH area			0.912
Variation explained by each PC after rotation	35.0	31.6	17.4

Extraction Method: Principal Component Analysis; Rotation Method: Varimax with Kaiser Normalization; Factors with loading coefficients < 0.70 have been suppressed

*MOH* Medical officer of health, *PC* Principal components, *PHI* Public health inspector

### Climate change vulnerability index

**Fig. 8:**
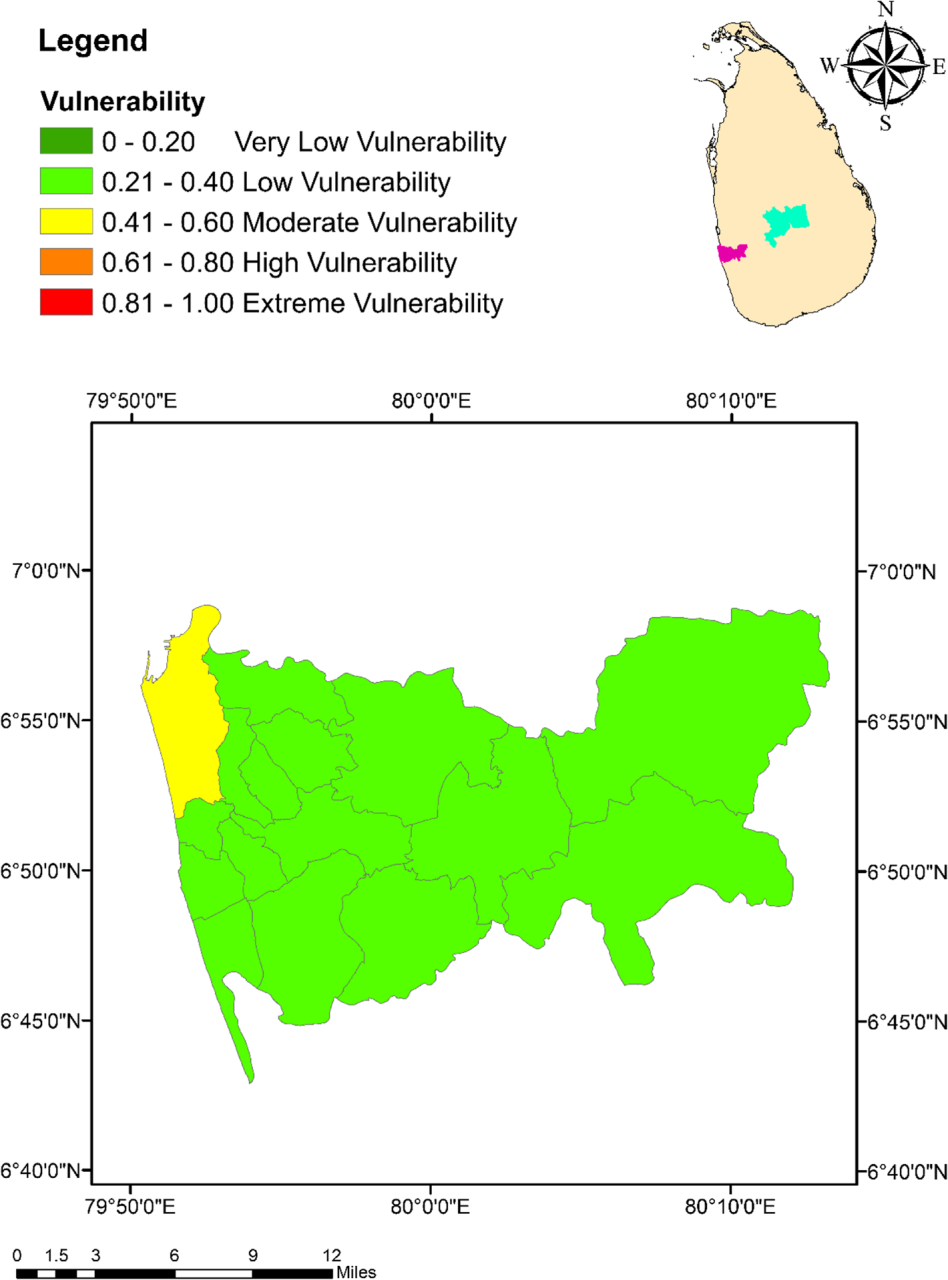
Spatial variation of the vulnerability of dengue to climate change among the MOH areas within the district of Colombo

**Fig. 9:**
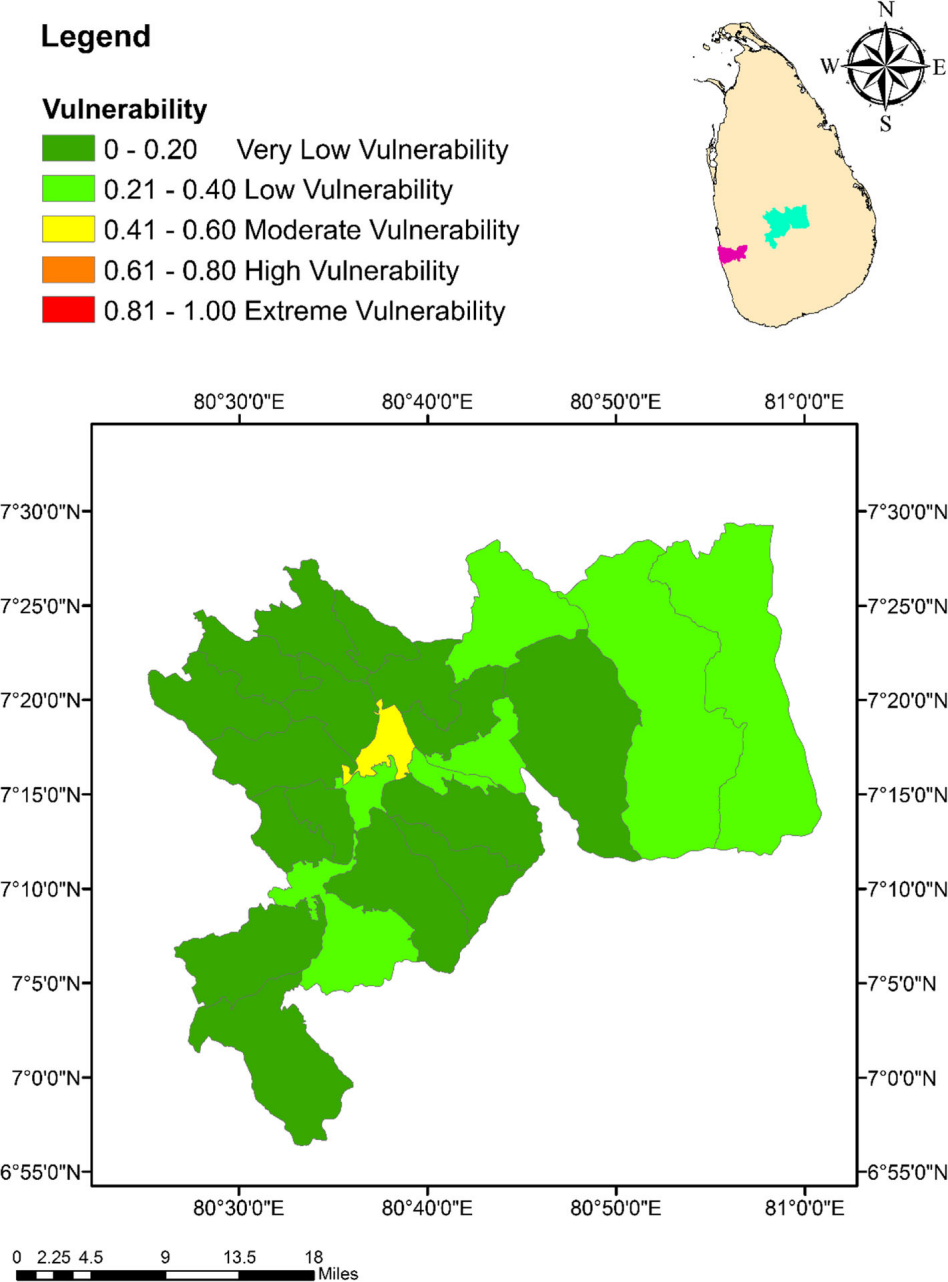
Spatial variation of the vulnerability of dengue to climate change among the MOH areas within the district of Kandy

The highest vulnerability of 0.45 (moderate vulnerability) was indicated by Colombo Municipal Council MOH area, while the lowest (0.15; very low vulnerability) was shown by Galaha MOH area in Kandy (Fig. 8). In general, the vulnerability index values of the MOH areas in Kandy (0.15 to 0.41) remained relatively lower than that of Colombo (0.31 to 0.45). However, it was interesting to note that Kandy Municipal Council MOH area had a vulnerability of 0.41 (moderate vulnerability), which was the highest vulnerability among the 23 MOH areas in Kandy as shown in Fig. 9.

## Discussion

Despite the complex interplay of multiple factors that influence the incidence of dengue, meteorological parameters play a vital role in influencing the timing and magnitude of dengue epidemics [17, 46]. With the limited success achieved during controlling dengue epidemics, recognition of vulnerable communities and evaluating the degree of vulnerability to dengue due to climate change is of paramount importance, especially in developing countries. This would also enable the implementation of proactive programmes to reduce existing vulnerabilities and to improve the resilience capacity of the vulnerable communities, guaranteeing the success of dengue epidemic management [17, 18].

### Exposure index (EI)

The EI considers the climate related hazardous events or trends and their direct physical impacts that impose a risk on dengue [31]. Monthly cumulative rainfall, average temperature and mean relative humidity retained as the climate related parameters in the EI, along with reported dengue cases, BI and PI as the direct physical impacts of climate variables. The rainfall events indicated a positive impact on the abundance of *Aedes* vectors by increasing the abundance of potential vector breeding sites either by replenishing water levels or formation of new breeding sites [22, 24], and modifying the relative humidity to favourable levels for mosquito survival and longevity [27]. However, extreme rainfall events followed by flooding may flush the *Aedes* larvae from their breeding sites resulting in a negative impact on the vector abundance [46]. Therefore, rainfall plays a key role in governing the population dynamics of *Aedes* vectors mosquitoes, allowing it to be considered as risk factor for increasing the exposure of dengue.

Relative humidity is another vital factor, which directly enhance the feeding frequency, inter sexual attractions and oviposition rates of *Aedes* mosquitoes [28]. Further, the adult longevity and survival success after being infected by DENV have also been found to increase under high humid conditions [27, 47] leading to a wide geographical dispersion of dengue [20]. In addition, higher levels of humidity have shown elevations in the duplication process of dengue fever, increasing the chance of DENV transmission [27, 48, 49].

On the other hand, temperature also cause favourable impacts on the incidence of dengue epidemics in several ways such as increasing the survival rate, accelerating the maturity rate and by shortening the EIP of DENV [22, 27, 49]. The average EIP of DENV was 12 days at 30 °C, which may be shortened to 7 days at 32 to 35 °C, resulting in higher transmission rates [50, 51]. *Aedes* larvae can survive at 34 °C water temperature, while the adults can survive even at 40 °C atmospheric temperature. Therefore, minimum temperature has been recognized as the limiting factor of *Aedes* population growth in many regions [20, 47]. Hence, global warming would favour higher levels of vector breeding and increase the abundance of *Aedes* mosquitoes leading to elevated risk levels of dengue. In addition, increased temperature due to global warming may increase the DENV transmission rates, which in turn increase the vulnerability of communities to dengue infection [46].

Despite the limitations and lapses in the entomological and epidemiological databases in Sri Lanka, the BI, PI and the number of reported dengue cases are the only reflective parameters of the direct impacts of climate variability on dengue [18]. Like many countries in the world, BI and PI are the most representative *stegomyia* indices that reflect the dynamics of dengue vector populations in Sri Lanka with an adequate accuracy [18, 52–54]. All vector controlling activities conducted by local VCE, are often guided by the BI, PI and the reported dengue cases, especially in timing the control efforts and in prioritizing the areas for resource allocation [18]. The current vulnerability assessment has recognized all these parameters under exposure, due to their capability of representing the direct physical impacts of climate variability on dengue within the studied MOH areas.

### Sensitivity index (SI)

The attributes that make the communities residing in Colombo and Kandy districts vulnerable to dengue under climate change, were considered under the SI [31]. Total population, percentage of males and females, percentage of population belonging to the age group of 21 to 40 years and above 60 years were selected as demographic parameters that reflect the sensitivity of local communities to dengue. As emphasized by previous studies, total population has often been recognized as a risk factor that increase the dengue risk. Among different age groups, only the proportion of population that is belonging to the age groups of 21 to 40 and > 60 years, were specifically recognized as groups with a relatively higher susceptibility to dengue in the PCA. Spending a relatively higher time duration at public places (such as work places, schools and public transport stations etc.) with elevated chances of being exposed to the bites of dengue vectors are potential reasons for the higher susceptibility of the people belonging to the age group of 20 to 40 years [19]. On the other hand, relatively lower immune strength to resist the DENV could be the contributing factor behind the high risk indicated by the elderly groups (> 60 years) in the considered communities [55].

Percentage of households practicing composting, disposing waste to the Municipal Council or Pradeshiya Sabha and burning waste also remained under sensitivity after the PCA. Properly planned urbanization and waste disposal services are key features that reduce the risk of dengue incidence in many countries [56, 57]. Maintaining solid waste for a long time, was found to enhance the breeding of *Aedes* mosquitoes [56]. Composting, collection of waste by the Municipality or Urban Council and burning of waste would essentially reduce the number of disposed containers aiding the source reduction of breeding sites [56]. Therefore, above factors heavily contribute to the reduction of the existing vulnerability of local communities to dengue.

Meanwhile, the extent of land covered by built-up environment and the forests were also included under the SI. The high prevalence of built-up (urban) environment is a critical risk factor associated with the incidence of dengue in many developing countries including Sri Lanka [19, 58, 59]. Meanwhile, forest areas could also provide ideal breeding (leaf axils and tree holes) and resting grounds, especially for *Ae. albopictus*, the secondary vector of dengue [58, 60].

### Adaptive capacity index (AC)

The knowledge possessed by the communities of potential technologies and methods for dengue control, institutional policies and resources owned by VCE for dengue management, that could be utilized to reduce the risk imposed by dengue, are considered under AC of the community [44]. Telecommunication facilities are widely used as an effective tool for awareness raising and community mobilization in the fight against dengue [60–62]. Often, television and radio have signified their vital importance in knowledge dissemination (regarding vector control and patient management), while allowing the VCE to motivate the local communities to contribute toward community involved dengue control activities [62, 63]. Therefore, the percentage of households with access to television and radios is a vital parameter that would enhance the adaptive capacity of local communities.

Population with no formal education or people with poor literacy, often act as a significant risk factor, since their awareness on the general vector management aspects and dengue control aspects remain limited [62–64]. On the contrary, people with a formal education level would be highly effective in community driven vector control activities conducted within the country, since they tend to share a higher level of knowledge on dengue along with positive attitudes toward supporting the VCE in the fight against dengue [19, 64]. The number of medical officers and PHI for 1000 residents are two of the vital indicators, which directly influence the health-related vulnerabilities of a community. In this case, both factors contributed to AC, due to their paramount importance in patient management aspects of dengue. Therefore, the availability of more medical officers and PHI would directly contribute to an elevated level of adaptive capacity, reducing the vulnerability of local communities to dengue.

### Vulnerability index (VI)

The relatively higher EI and SI values in the Colombo district (0.71–0.89 and 0.38–0.86, respectively) than in Kandy (0.19–0.79 and 0.15 to 0.77) could be the reason behind the spatial disparity of the composite VI. When the AC values are considered, the MOH areas in Kandy denoted a relatively higher AC (0.68–0.16) level than Colombo (0.66–0.28). This also plays a significant role in relatively higher VI levels for the district of Colombo. The MOH areas in Colombo are characterized by high rate of urbanization, poorly planned infrastructure facilities (especially waste disposal), high population densities, prevalence of notable levels of slums and built-up environments, which could have caused elevated SI levels. Meanwhile, the relatively higher temperature, high levels of BI and PI could lead to relatively higher EI levels in Colombo [19, 56, 64]. On the other hand, the district of Kandy is characterized by relatively low level of BI and PI, degree of urbanization, population density and built environment [18, 55]. Variations in the degree of urbanization and the environmental characteristics among these two districts could be recognized as the influencing factors for spatial dissimilarities in VI [19, 55]. In both districts the urban centers, namely CMC and KMC MOH areas denoted notable levels of vulnerabilities, further supporting the above claim.

On the other hand, previous studies in these two districts suggest that the local communities in Kandy have relatively higher level of awareness on dengue, with better attitudes towards dengue control [19]. Further, the environmental management and dengue preventive practices have also been better among the local communities in Kandy, than in Colombo [19, 55]. This can also play a critical role in influencing the climate change induced vulnerability of local communities to dengue, as such parameters may cause direct and indirect impacts on EI, SI and AC. Meanwhile, a relatively higher spatial variation in VI was observed within the Kandy district, which may also be attributed to the diverse nature in the degree of urbanization (rural to urban), land use and socio-economic conditions described above under individual indices [19].

Proper identification of the risk factors that directly characterize the risk imposed by dengue outbreaks, understanding the relationship of those factors with dengue outbreak incidence and evaluation of the vulnerability of local communities to dengue, are key requirements in understanding the actual burden of dengue on the country [17]. The current study lacked long term data on the seroprevalence of dengue viruses and more reliable entomological parameters such as pupal index data, which could have provided more conclusive results. In addition, authors had to rely upon a limited number of meteorological stations in the study area, due to physical limitations of the country. Despite above limitations, recognition of the most vulnerable localities and their potential risk factors would immensely assist the VCE in implementing proactive programs to reduce existing vulnerabilities and enhancing resilience capacity of the vulnerable communities, to ensure the success of dengue epidemic management [17, 18]. In addition, the findings would enable the VCE to remain prepared for the expected variations in dengue risk caused by the changing climate.

## Conclusions

Among 42 interrelated variables, a total of six EI variables, 11 SI variables and six AC variables were selected to assess the climate change vulnerability of dengue in the districts of Colombo and Kandy. Colombo Municipal Council MOH area denoted the highest vulnerability (0.46: moderate vulnerability) to dengue, while the Galaha MOH showed the lowest (0.15; very low vulnerability). In general, the vulnerability for dengue was relatively higher within the MOH areas of Colombo, than in Kandy, suggesting a higher degree of potential susceptibility to dengue within the local communities of Colombo. Interestingly the KMC MOH area had a vulnerability of 0.41 (moderate vulnerability), which was the highest within Kandy. The high degree of urbanization, poorly planned infrastructure facilities (especially waste disposal), notable levels of slums and shanties and high percentage of built-up environments along with relatively higher temperature could be recognized as the key factors that have caused elevated VI levels. Meanwhile, better attitudes and practices (environmental management and dengue preventive) among local communities could notably reduce the climate change induced vulnerability of local communities to dengue. Therefore, the VCE are recommended to consider the spatial variations along with above driving factors in decision making to manage the vulnerability of local communities to dengue, especially in allocation of limited financial, human and mechanical resources for dengue epidemic management.

## Data Availability

The datasets supporting the conclusions of this article are included within the article.

## Abbreviations

AC: Adaptive capacity index
BI: Breteau index
BI_agp_: Breteau index for *Ae. aegypti*
CMC: Colombo Municipal Council
KMC: Kandy Municipal Council
EI: Exposure index
MOH: Medical Officer of Health
PC: Principal component
PCA: Principal component analysis
PI: Premises index
SI: Sensitivity index
VCE: Vector controlling entities
VI: Vulnerability index

## References

[1] Bhatt S, Gething PW, Brady OJ, Messina JP, Farlow AW, Moyes CL, Drake JM, Brownstein JS, Hoen AG, Sankoh O, Myers MF (2013). The global distribution and burden of dengue. Nature..

[2] Halasa YA, Shepard DS, Zeng W (2012). Economic cost of dengue in Puerto Rico. Am J Trop Med Hyg..

[3] Githeko AK (2012). Advances in developing a climate based dengue outbreak models in Dhaka, Bangladesh: challenges & opportunities. Indian J Med Res.

[4] Walker KR, Joy TK, Ellers-Kirk C, Ramberg FB (2011). Human and environmental factors affecting *Aedes aegypti* distribution in an arid urban environment. J Am Mosq Control Assoc.

[5] Thai KT, Anders KL (2011). The role of climate variability and change in the transmission dynamics and geographic distribution of dengue. Exp Biol Med.

[6] Pinto E, Coelho M, Oliver L, Massad E (2011). The influence of climate variables on dengue in Singapore. Int J Environ Health Res.

[7] World Health Organization, Special Programme for Research, Training in Tropical Diseases, World Health Organization. Department of Control of Neglected Tropical Diseases, World Health Organization. Epidemic, Pandemic Alert. Dengue: guidelines for diagnosis, treatment, prevention and control. France: World Health Organization; 2009.

[8] Teixeira MD, Barreto ML, Costa MD, Ferreira LD, Vasconcelos PF, Cairncross S (2002). Dynamics of dengue virus circulation: a silent epidemic in a complex urban area. Tropical Med Int Health.

[9] Gubler DJ (2002). Epidemic dengue/dengue hemorrhagic fever as a public health, social and economic problem in the 21st century. Trends Microbiol.

[10] Githeko AK, Lindsay SW, Confalonieri UE, Patz JA (2000). Climate change and vector-borne diseases: a regional analysis. Bull World Health Organ.

[11] Epidemiology Unit, Ministry of Health, Sri Lanka (2019). Dengue update.

[12] Gubler DJ (1998). Resurgent vector-borne diseases as a global health problem. Emerg Infect Dis.

[13] Sutherst RW (2004). Global change and human vulnerability to vector-borne diseases. Clin Microbiol Rev.

[14] Kovats RS, Campbell-Lendrum DH, McMichel AJ, Woodward A, Cox JS (2001). Early effects of climate change: do they include changes in vector-borne disease?. Philos Trans R Soc Lond B Biol Sci.

[15] McMichael AJ, Woodruff RE, Hales S (2006). Climate change and human health: present and future risks. Lancet..

[16] Hales S, De Wet N, Maindonald J, Woodward A (2002). Potential effect of population and climate changes on global distribution of dengue fever: an empirical model. Lancet..

[17] Hagenlocher M, Delmelle E, Casas I, Kienberger S (2013). Assessing socioeconomic vulnerability to dengue fever in Cali, Colombia: statistical vs expert-based modeling. Int J Health Geogr.

[18] Udayanga L, Gunathilaka N, Iqbal MC, Najim MM, Pahalagedara K, Abeyewickreme W (2018). Empirical optimization of risk thresholds for dengue: an approach towards entomological management of *Aedes* mosquitoes based on larval indices in the Kandy District of Sri Lanka. Parasit Vectors.

[19] Udayanga L, Gunathilaka N, Iqbal MC, Lakmal K, Amarasinghe US, Abeyewickreme W (2018). Comprehensive evaluation of demographic, socio-economic and other associated risk factors affecting the occurrence of dengue incidence among Colombo and Kandy districts of Sri Lanka: a cross-sectional study. Parasit Vectors.

[20] Promprou S, Jaroensutasinee M, Jaroensutasinee K (2005). Climatic factors affecting dengue haemorrhagic fever incidence in southern Thailand. Dengue Bull.

[21] Harrington LC, Buonaccorsi JP, Edman JD, Costero A, Kittayapong P, Clark GG, Scott TW (2001). Analysis of survival of young and old *Aedes aegypti* (Diptera: Culicidae) from Puerto Rico and Thailand. J Med Entomol.

[22] Lindsay M, Mackenzie J (1997). Vector-borne viral diseases and climate change in the Australasian region: major concerns and the public health response. Climate change and human health in the Asia-Pacific region: Australian Medical Association and Greenpeace International.

[23] Jansen CC, Beebe NW (2010). The dengue vector *Aedes aegypti*: what comes next?. Microbes Infect.

[24] Gubler DJ, Reiter P, Ebi KL, Yap W, Nasci R, Patz JA (2001). Climate variability and change in the United States: potential impacts on vector-and rodent-borne diseases. Environ Health Perspect.

[25] Woodruff RE, Guest CS, Garner MG, Becker N, Lindesay J, Carvan T, Ebi K. Predicting Ross River virus epidemics from regional weather data. Epidemiology. 2002;13(4):384–93. 10.1097/00001648-200207000-00005 12094092

[26] Wu PC, Guo HR, Lung SC, Lin CY, Su HJ (2007). Weather as an effective predictor for occurrence of dengue fever in Taiwan. Acta Trop.

[27] McMichael AJ, Haines A, Slooff R, Kovats S (1996). Climate change and human health.

[28] Rowley WA, Graham CL (1968). The effect of temperature and relative humidity on the flight performance of female *Aedes aegypti*. J Insect Physiol.

[29] Thu HM, Aye KM, Thein S (1998). The effect of temperature and humidity on dengue virus propagation in *Aedes aegypti* mosquitos. Se Asian J Trop Med.

[30] Intergovernmental Panel on Climate Change (2007). Climate change. Contribution of working group I to the fourth assessment report of the intergovernmental panel on climate change. The physical science basis.

[31] Parry ML, Canziani O, Palutikof J, Van der Linden P, Hanson C (2007). Climate change 2007-Impacts, adaptation and vulnerability: Working group II contribution to the fourth assessment report of the IPCC: Cambridge University Press.

[32] Smit B, Wandel J (2006). Adaptation, adaptive capacity and vulnerability. Global Environ Chang.

[33] Brooks N, Adger WN, Kelly PM (2005). The determinants of vulnerability and adaptive capacity at the national level and the implications for adaptation. Global Environ Chang..

[34] Birkmann J. Measuring vulnerability to towards disaster resilient societies. Japan: Nations University Press; 2006.

[35] Cutter SL, Boruff BJ, Shirley WL (2003). Social vulnerability to environmental hazards. Soc Sci Q.

[36] de Mattos Almeida MC, Caiaffa WT, Assunçao RM, Proietti FA (2007). Spatial vulnerability to dengue in a Brazilian urban area during a 7-year surveillance. J Urban Health.

[37] Martinez TTP, Rojas LI, Valdés LS, Noa RR (2003). Spatial vulnerability to dengue: an application of the geographic information systems in Playa municipality, City of Havana. Rev Cub Salud Publica.

[38] Tipayamongkholgul M, Lisakulruk S (2011). Socio-geographical factors in vulnerability to dengue in Thai villages: a spatial regression analysis. Geospat Health.

[39] Chang AY, Parrales ME, Jimenez J, Sobieszczyk ME, Hammer SC, Copenhaver DJ, Kulkarni RP (2009). Combining Google earth and GIS mapping technologies in a dengue surveillance system for developing countries. Int J Health Geogr.

[40] Vanwambeke SO, van Benthem BHB, Khantikul N, Burghoorn-Maas C, Panart K, Oskam L, Lambin EF, Pradya (2006). Multi-level analyses of spatial and temporal determinants for dengue infection. Int J Health Geogr.

[41] Vazquez-Prokopec GM, Stoddard ST, Paz-Soldan V, Morrison AC, Elder JP, Kochel TJ, Scott TW, Kitron U (2009). Usefulness of commercially available GPS data-loggers for tracking human movement and exposure to dengue virus. Int J Health Geogr.

[42] Colombo District Secretariat, Sri Lanka. 2018. http://www.colombo.dist.gov.lk. Accessed 16 Nov 2019.

[43] Kandy District Secretariat, Sri Lanka. 2018. http://www.kandy.dist.gov.lk. Accessed 25 Nov 2019.

[44] Fritzsche K, Schneiderbauer S, Bubeck P, Kienberger S, Buth M, Zebisch M, Kahlenborn W (2014). The vulnerability sourcebook: concept and guidelines for standardised vulnerability assessments: Verlag Nicht Ermittelbar.

[45] Abson DJ, Dougill AJ, Stringer LC (2012). Using principal component analysis for information-rich socio-ecological vulnerability mapping in southern Africa. Appl Geogr.

[46] Alkhaldy I (2017). Modelling the association of dengue fever cases with temperature and relative humidity in Jeddah, Saudi Arabia—a generalised linear model with break-point analysis. Acta Trop.

[47] Christophers SR (1960). The yellow fever mosquito. Its Life History, Bionomics and Structure.

[48] Hales S, Weinstein P, Souares Y, Woodward A (1999). El Niño and the dynamics of vectorborne disease transmission. Environ Health Perspect.

[49] Barbazan P, Yoksan S, Gonzalez JP (2002). Dengue hemorrhagic fever epidemiology in Thailand: description and forecasting of epidemics. Microbes Infect.

[50] Focks DA, Daniels E, Haile DG, Keesling JE (1995). A simulation model of the epidemiology of urban dengue fever: literature analysis, model development, preliminary validation, and samples of simulation results. Am J Trop Med Hyg..

[51] Watts DM, Burke DS, Harrison BA, Whitmire RE, Nisalak A (1987). Effect of temperature on the vector efficiency of *Aedes aegypti* for dengue 2 virus. Am J Trop Med Hyg..

[52] Pontes RJ, Freeman JO, Oliveira-Lima JW, Hodgson JC, Spielman AN (2000). Vector densities that potentiate dengue outbreaks in a Brazilian city. Am J Trop Med Hyg.

[53] Sanchez L, Vanlerberghe V, Alfonso L, del Carmen MM, Guzman MG, Bisset J, Van Der Stuyft P (2006). *Aedes aegypti* larval indices and risk for dengue epidemics. Emerg Infect Dis.

[54] Abeyewickreme W, Wickremasinghe AR, Karunatilake K, Sommerfeld J, Axel K (2012). Community mobilization and household level waste management for dengue vector control in Gampaha district of Sri Lanka; an intervention study. Pathog Glob Health.

[55] Udayanga L, Gunathilaka N, Iqbal MC, Pahalagedara K, Amarasinghe US, Abeyewickreme W (2018). Socio-economic, knowledge attitude practices (KAP), household related and demographic based appearance of non-dengue infected individuals in high dengue risk areas of Kandy District, Sri Lanka. BMC Infect Dis.

[56] Gubler DJ, Clark GG (1996). Community involvement in the control of *Aedes aegypti*. Acta Trop.

[57] Cheong YL, Leitão PJ, Lakes T (2014). Assessment of land use factors associated with dengue cases in Malaysia using boosted regression trees. Spat Spatio-temporal Epidemiol.

[58] Toha HR, Hashim JH, Sahani M, Shamsir MS. Spatial occurrence of dengue fever and its relationship with land use in Selangor, Malaysia. BMC Public Health. 2014;14(Suppl 1):16.

[59] Lee HL (1991). A nationwide resurvey of the factors affecting the breeding of *Aedes aegypti* (L.) and *Aedes albopictus* (Skuse) (Diptera: Culicidae) in urban town of peninsular Malaysia–1988–1989. Trop Biomed.

[60] Pérez-Guerra CL, Seda H, García-Rivera EJ, Clark GG (2005). Knowledge and attitudes in Puerto Rico concerning dengue prevention. Rev Panam Salud Publica.

[61] Itrat A, Khan A, Javaid S, Kamal M, Khan H, Javed S, Kalia S, Khan AH, Sethi MI, Jehan I (2008). Knowledge, awareness and practices regarding dengue fever among the adult population of dengue hit cosmopolitan. PLoS One.

[62] Banneheke H, Paranavitane S, Jayasuriya V, Banneheka S (2016). Perceived risk of dengue in ones’ living environment as a determinant of behavior change through social mobilization and communication: evidence from a high-risk area in Sri Lanka. J Arthropod Borne Di.

[63] Gunasekara TD, Velathanthiri VG, Weerasekara MM, Fernando SS, Peelawattage M, Guruge D, Fernando S (2015). Knowledge, attitudes and practices regarding dengue fever in a suburban community in Sri Lanka. Galle Med J.

[64] Alobuia WM, Missikpode C, Aung M, Jolly PE (2015). Knowledge, attitude, and practices regarding vector-borne diseases in Western Jamaica. Ann Glob Health.

